# Improved Identification of Rapidly Growing Mycobacteria by a 16S–23S Internal Transcribed Spacer Region PCR and Capillary Gel Electrophoresis

**DOI:** 10.1371/journal.pone.0102290

**Published:** 2014-07-11

**Authors:** Timothy J. Gray, Fanrong Kong, Peter Jelfs, Vitali Sintchenko, Sharon C-A. Chen

**Affiliations:** 1 Centre for Infectious Diseases and Microbiology, Institute of Clinical Pathology and Medical Research, Westmead, New South Wales, Australia; 2 Centre for Infectious Diseases and Microbiology – Public Health, Westmead Hospital, Westmead, New South Wales, Australia; 3 Marie Bashir Institute for Emerging Infections and Biosecurity, Sydney Medical School, The University of Sydney, Sydney, New South Wales, Australia; National Institute of Infectious Diseases, Japan

## Abstract

The identification of rapidly growing mycobacteria (RGM) remains problematic because of evolving taxonomy, limitations of current phenotypic methods and absence of a universal gene target for reliable speciation. This study evaluated a novel method of identification of RGM by amplification of the mycobacterial 16S–23S rRNA internal transcribed spacer (ITS) followed by resolution of amplified fragments by capillary gel electrophoresis (CGE). Nineteen American Type Culture Collection (ATCC) *Mycobacterium* strains and 178 clinical isolates of RGM (12 species) were studied. All RGM ATCC strains generated unique electropherograms with no overlap with slowly growing mycobacteria species, including *M. tuberculosis*. A total of 47 electropherograms for the 178 clinical isolates were observed allowing the speciation of 175/178 (98.3%) isolates, including the differentiation of the closely related species, *M. massiliense* (*M. abscessus* subspecies *bolletii*) and *M. abscessus* (*M. abscessus* sensu stricto). ITS fragment size ranged from 332 to 534 bp and 33.7% of clinical isolates generated electropherograms with two distinct peaks, while the remainder where characterized with a single peak. Unique peaks (fragment lengths) were identified for 11/12 (92%) RGM species with only *M. moriokaense* having an indistinguishable electropherogram from a rarely encountered CGE subtype of *M. fortuitum*. We conclude that amplification of the 16S–23S ITS gene region followed by resolution of fragments by CGE is a simple, rapid, accurate and reproducible method for species identification and characterization of the RGM.

## Introduction

Rapidly growing mycobacteria (RGM) are ubiquitous environmental acid-fast bacilli that are important human and animal pathogens and are characterised by their growth in culture medium within 7 days post-inoculum. They cause infection in both immunocompetent and immunocompromised individuals, most commonly manifesting as pulmonary or skin and soft tissue infection, and less frequently as a cause of bacteremia, osteomyelitis, endocarditis, peritonitis, lymphadenitis, post-surgical infections, or catheter-related infection [1], [2]. The speciation of the RGM facilitates the selection of the appropriate antimicrobial therapy, may predict disease progression and allows for the detection of novel species. In addition, speciation allows for the initial recognition of epidemiologically-linked cases that have been reported to occur in hospitals and in the community, with public health implications [3], [4].

Historically, a combination of phenotypic biochemical and mycolic acid-based identification methods have been used to identify clinically relevant RGM, but these techniques are slow, lack discriminatory power to distinguish closely-related species and cannot identify novel species. Proteomic approaches, including matrix assisted laser desorption ionization time-of-flight mass spectrometry (MALDI-TOF MS), have been reported to speciate RGM; however, the ability of this technology to reliably distinguished closely related species remains uncertain [5]–[7]. Molecular methods include probe-based assays, PCR restriction enzyme analysis and gene sequence analysis, and have emerged as attractive approaches for species identification of mycobacteria [8]. Molecular targets that have been used include the 16S rRNA gene, the heat shock protein 65 (*hsp*65) gene, the β subunit of the RNA polymerase (*rpoB*) gene, as well as the 16S–23S internal transcribed spacer (ITS) [9]. The ITS region has been shown to be of particular utility in taxonomic assignment of mycobacteria because it is less highly conserved compared with the 16S rRNA gene and has potential to yield a greater degree of genetic polymorphism [9]–[11]. Yet no single gene target, including the ITS, has been shown to be sufficiently discriminatory to speciate all RGM [9].

The most commonly encountered RGM are species within the *Mycobacterium fortuitum* complex and the *M. chelonae* -*abscessus* group [1], [2]. Before 2001 only two species, *M. chelonae* and *M. abscessus*, were recognised within the *M. chelonae-abscessus* group, however, more recently division of the *M. abscessus* group has been suggested. Some authors propose that *M. abscessus* be subdivided into three species - *M. abscessus*, *M. massiliense* and *M. bolletii* [12], [13]. Others have indicated that these three organisms represent two subspecies of the *M. abscessus* group – ie. *M. abscessus* sensu stricto and *M. abscessus* subspecies *bolletii* – with the latter incorporating both *M. massiliense* and *M. bolletii* [14]. Importantly, *M. abscessus*, *M. massiliense* and *M. bolletii* cannot be distinguished using traditional phenotypic methods [14] and all three organisms share 100% sequence identity of the mycobacterial 16S rRNA gene [14], [15]. Yet, clinical evidence has emerged that separating *M. abscesses* from *M. massiliense* is potentially useful as these organisms are associated with different disease phenotypes and progression [16]–[18]. In addition, inducible macrolide resistance is commonly encountered in *M. abscessus* sensu stricto, which harbours an intact erythromycin ribosomal methylase (*erm*) gene, while *M. massiliense* isolates are stably susceptible to macrolides as a result of two deletions within the *erm* gene [19], [20]. The species names *M. abscessus* (*M. abscessus* sensu stricto) and *M. massiliense* (*M. abscessus* subspecies *bolletii*) continue to dominate the clinical literature and this is the terminology used within this study. Speciation of members within the *M. fortuitum* complex is seldom performed since the clinical utility of this practice is uncertain and because existing methods are time consuming and expensive [8].

In this study, we have developed and evaluated a method to identity RGM based on amplification of the *Mycobacteria* 16S–23S rRNA ITS region(s) followed by amplicon analysis by sequencer-based capillary gel electrophoresis (CGE). Unlike traditional gel electrophoresis, CGE uses 5′-end fluorescein-labelled primers and a DNA analyzer, with rapid, accurate resolution of amplicons. It has shown good discriminatory power in the identification and characterization of other clinically significant bacteria including *Clostridium difficile* [21], [22], *Vibrio* [23], and *Nocardi*a species [24]. We applied this method to a representative set of clinical isolates identified by a combination of HPLC, real time PCR and 16S rRNA gene sequencing. The antimicrobial profiles of the *M. abscessus* group study isolates, where available, were also reviewed to investigate possible association, if any, between CGE profiles and antibiotic susceptibility.

## Methods

### Isolates

All RGM isolates referred to the New South Wales Mycobacterium Reference Laboratory, Westmead Hospital, Sydney, Australia between 1 March 2012 and 31 January 2013 were included in the study (n = 213). Duplicate isolates cultured from the same patient (n = 26), contaminated isolates (n = 6) and missing isolates (n = 6) were excluded. An additional 3 organisms (isolated after December 2011) representing uncommon RGM species were also retrieved. Study isolates were stored on Middlebrook 7H11 agar at 4°C. Nineteen American Type Culture Collection strains (ATCC, Manassas, VA) mycobacterial strains (4 RGM and 15 slowly-growing organisms) were also studied as shown in Table 1.

**Table 1:** American Type Culture Collection (ATCC) strains that underwent capillary gel electrophoresis of the 16S–23S internal transcribed spacer region with results rounded to the nearest bp.

Species/Type Strain		Fragment 1 (bp)	Fragment 2 (bp)
***Rapidly Growing Mycobacteria***			
*M. chelonae*	ATCC 35752	384	
*M. abscessus*	ATCC 19977	388	
*M. fortuitum*	ATCC 6841	435	458
*M. mucogenicum*	ATCC 49650	521	523
***Mycobacterium tuberculosis complex***			
*M. tuberculosis*	ATCC 27294	364	
***Slowly growing Mycobacteria***			
*M. gordonae*	ATCC 14470	362	
*M. kansasii*	ATCC 12478	364†	
*M. marinum*	ATCC 927	364†	
*M. szulgai*	ATCC 35799	365‡	
*M. shimodei*	ATCC 27962	365‡	
*M. avium*	ATCC 25291	367	369
*M. asiaticum*	ATCC 25276	369	
*M. paraffinicum*	ATCC 12670	370	
*M. scrofulaceum*	ATCC 19981	370	372
*M. intracellulare*	ATCC 13950	371	
*M. simiae*	ATCC 25275	373	
*M. xenopi*	ATCC 19250	448	
*M. nonchromogenicum*	ATCC 19530	453	
*M. terrae*	ATCC 15755	456	

† *M. kansasii* and *M. marinum* were distinguished using HPLC [25].

‡ *M. szulgai* and *M. shimodei* were distinguished using HPLC [25].

### Identification of Mycobacteria

RGM were initially characterized by high-performance liquid chromatography (HPLC) analysis as previously described [25]. Isolates with HPLC profiles consistent with *M. chelonae*/*M. abscessus* group were confirmed by an in-house real-time multiplex PCR assay, using primers and species-specific TaqMan probes targeting the ITS region of the 16S–23S rRNA gene, adapted from the method of Xiong *et al.* [26]. Isolates identified as *M. abscessus* were then further speciated to *M. abscessus* and *M. massiliense* by sequencing the full ITS segment (primers described below) with reference to published sequences (GenBank Accession no. CU458896; GenBank Accession no. CP003699). Other study isolates, including *M. fortuitum* complex, *M. mucogenicum*, *M. flavescens*, *M. elephantis*, *M. immunogenum*, *M. moriokaense*, *M. septicum*, *M. neoaurum* and *M. phlei* were identified to species level using a combination of HPLC profile analysis followed by 16S rRNA sequencing when atypical HPLC traces were encountered (Table S1 and Figure S1) [27]. The 16S rRNA gene sequences of isolates were compared with sequences of type strains archived in the NCBI GenBank using NCBI BLASTn algorithm (www.ncbi.nlm.nih.gov/GenBank/).

### DNA extraction

Bacterial DNA was extracted using the commercial InstaGene Matrix method (Bio-Rad Laboratories Inc, California, USA). Briefly, 2–4 individual mycobacterial colonies were placed into 1 mL of sterile water, mixed by vortex and then underwent sonication for 20 minutes. Suspensions were centrifuged at 11,000 rpm for 1 minute after which the supernatant was discarded. The residual pellet was resuspended in 200 µL of InstaGene Matrix (Bio-Rad), mixed by vortex and incubated at 56°C for 20 minutes. Samples were then boiled for 8 minutes and centrifuged (13,200 rpm for 2 minutes) prior to use. Samples were used within 24 hours or stored at −20°C. Routine protocols in our laboratory have demonstrated minimal degradation of extracted DNA under these conditions (data not shown).

### Amplification of the 16S–23S rRNA internal transcribed spacer

The mycobacteria ITS region(s) were amplified prior to CGE analysis using a 6-FAM labelled ITS1F forward primer 5′ GTGCGGCTGGATCACCTCCT 3′ and a ITS1R reverse primer 5′ AGCCTCCYACGTCCTTC(A/T)TCGGCT 3′ (Sigma-Aldrich, Castle Hill, NSW, Australia). Sequencing of the ITS region was performed for *M. chelonae, M. abscessus* and *M. massiliense* using an ITS2F forward primer 5′ GAAGTCGTAACAAGGTAGCCG 3′ and a ITS2R reverse primer 5′ GACAGCTCCCCGAGGC(A/T)TATCGCA 3′ (Sigma-Aldrich). Each PCR mixture was made to a total volume of 25 µL consisting of 5 µL of DNA extract, 0.5 µM forward and reverse primers, 2.5 mM deoxynucleoside triphosphates (Roche, Castle Hill, Australia), 10× PCR buffer containing 15 mM MgCl_2_ (Qiagen, Doncaster, Victoria, Australia), 0.5 U HotStarTaq DNA polymerase (Qiagen) and molecular biology-grade H_2_O (Eppendorf, North Ryde, Australia). PCR conditions were 95°C for 15 minutes and then 35 cycles of 95°C for 30 seconds, 62°C for 30 seconds, 72°C for 1 minute, followed by a further extension at 72°C for 10 minutes.

### Sequencer-based capillary gel electrophoresis

PCR fragment analysis was performed using the ABI 3730xl DNA analyzer employing a 48-capillary 50 cm POP-7 gel (Applied Biosystems, Forster City, USA). PCR products were diluted 1∶30 with molecular biology-grade H_2_O (Eppendorf) to a final volume of 30 µl. Sample injection was at 1.6 kV over 15 seconds with a total running time of 6,200 seconds at 15 kV run voltage. A 20- to 1200-bp LIZ 1200 ladder (Chimerx, Madison, WI, USA) was used as an internal size control.

### Result interpretation, reproducibility testing and data analysis

Amplified fragments, represented by one or more peaks according to fragment size, were analysed by GeneMapper software (Applied Biosystems). Smaller peaks were required to reach 10% of the height of the greatest peak and when double peaks were observed ≤1.0 bp apart, only the larger peak was analysed [21], [22], [24]. Fragment lengths found in >10% of a species were called ‘common’ and fragment lengths found only in one species were defined as ‘unique’. Descriptive statistics were prepared using IBM SPSS Statistics 21.0 (IBM Corp., NY, USA). The pattern of peaks was noted in relation to the number and numerical value of each fragment rounded to the nearest whole number, with the exception of *M. abscessus* and *M. massiliense* isolates which were rounded to the nearest 0.5 bp.

A random set of the study DNA extracts (19/178, 10.7%), underwent repeat ITS-CGE testing to assess reproducibility. In addition, 3 patients with repeat isolates of RGM collected at different times during the study period (>1 month apart), had DNA extracted on each specimen with ITS-CGE results compared.

### Antibiotic Susceptibility testing

*M. abscessus* and *M. massiliense* isolates underwent susceptibility testing as determined by clinical need by previously described methods [28] using the Versa TREK system (formerly ESP II; Trek Diagnostic Systems, West Lake, OH). Antibiotics tested included co-trimoxazole, linezolid, ciprofloxacin, imipenem, moxifloxacin, cefepime, cefoxitin, ceftriaxone, amoxicillin-clavulanate, amikacin, doxycycline, micocycline, tigecycline, tobramycin and clarithromycin. Comparison of minimum inhibitory concentrations (MICs) was by the Mann-Whitney U-tests, performed using IBM SPSS Statistics 21.0 (IBM).

### Ethics Statement

This study is not a research question but describes a laboratory validation of a novel method for the specific identification of mycobacterial pathogens grown on artificial media. In addition, the study does not involve the collection or reporting of patient data and no patient intervention occurred with the obtained results. Bacterial isolates were collected and stored as part of routine laboratory practice. No additional isolates were collected or stored for the purpose of this study. All bacterial samples were de-identified for the purpose of study experiments, data analysis and reporting.

## Results

A total of 178 clinical isolates collected from different patients and representing 12 RGM were included in the study set.

### Testing of reference strains

The 4 RGM ATCC strains generated distinct ITS-CGE electropherograms when compared with the 15 slow growing mycobacteria ATCC strains, including *M. tuberculosis* (Table 1). All 4 RGM ATCC strains generated distinct peaks, with a measured difference of 4 bp separating *M. abscessus* (ATCC 1997) and *M. chelonae* (ATCC 35752). Of the 4 ATCC RGM strains, *M. abscessus* (ATCC 1997) and *M. chelonae* (ATCC 35752) had single peaks while *M. fortuitum* (ATCC 6841) and *M. mucogenicum* (ATCC 49650) had double peaks (Table 1). There were single indistinguishable peaks for *M. kansasii* (ATCC 12478) and *M. marinum* (ATCC 927) at 364 bp, and also for the *M. szulgai* (ATCC 35799) and *M. shimodei* (ATCC 27962) strains at 365 bp. In addition, ITS-CGE was unable to distinguish *M. tuberculosis* (ATCC 27294) from the *M. szulgai* and *M. shimodei* ATCC strains. These 5 slowly growing species were separated by traditional mycolic acid analysis using HPLC (data not shown) [25].

### Identification of Clinical Isolates

**Figure 1:**
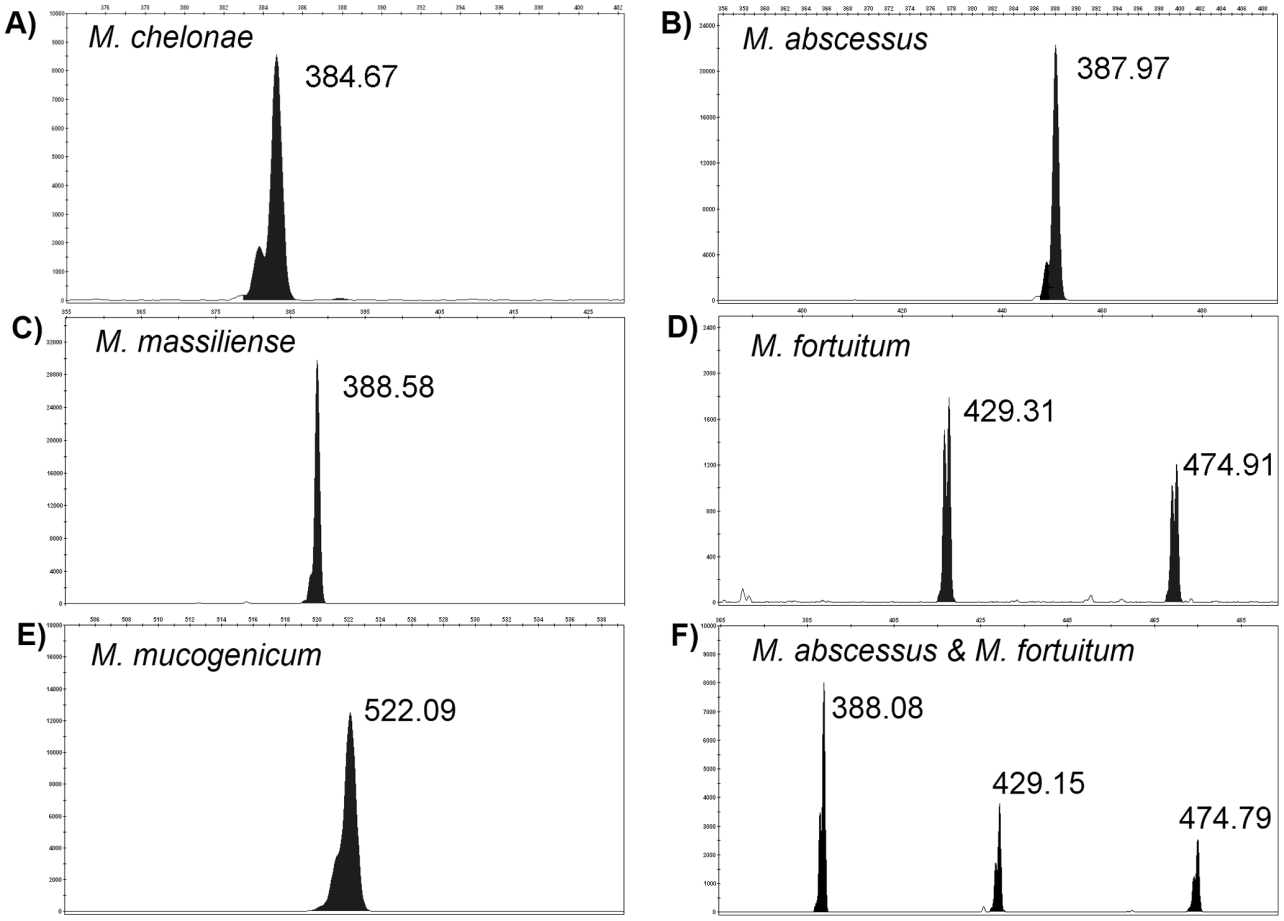
Representative CGE electropherograms following PCR amplification of the 16S–23S rRNA internal transcribed spacer (ITS) for A) *M. chelonae*; B) *M. abscessus*; C) *M. massiliense*; D) *M. fortuitum*; and E) *M. mucogenicum*. Peaks correlate with the ITS fragment length(s) which is shown above each peak. Panel A and D highlight the phenomenon of spurious double peaks which were less than 1 bp apart. Panel F shows the ITS-CGE electropherogram following the pre-extraction mix of *M. abscessus* and *M. fortuitum* demonstrating the typical peaks for each isolate.

ITS region amplicons were obtained for all 178 clinical isolates. CGE analysis demonstrated single peaks for 118 (66.3%) isolates and double peaks for 60 (33.7%) isolates; ITS fragment sizes ranged from 332 to 534 bp. Overall, CGE analysis generated 47 unique electropherograms for the 178 isolates. Figure 1 (Panel A–E) highlights representative examples of the electropherograms for the study isolates.

Unique single peak electropherograms were evident for all 31 *M. abscessus*, 17 *M. massiliense* and 40 *M. chelonae* isolates, separating these closely related species. There were two patterns generated for the *M. abscessus* species, one with average measured amplicon length 387.98 bp (range 387.74–388.15; n = 29) and a second with length of 393 bp (n = 2). Both of these unique electropherograms were distinguished from the 17 *M. massiliense* isolates which generated an average measured CGE fragment size of 388.70 bp (range 388.44–388.93). Sequencing of the ITS fragments revealed 3 consistent point mutations between the *M. abscessus* and the *M. massiliense* isolates, including a single deletion of a cytosine base, accounting for the small but distinguishable fragment length differences. The 2 *M. abscessus* isolates with fragment length of 393 bp were epidemiologically distinct. The isolates were cultured from the sputum of patients collected from different jurisdictions of Australia (>1500 km apart). Sequencing of the ITS region revealed both isolates carried a 5 bp insertion polymorphism (-TTGTG-) not described in GenBank. Further sequencing of the full 16S gene for these two isolates demonstrated it was homologous with published sequences of *M. abscessus/M. massiliense*, (GenBank Accession no. CU458896; GenBank Accession no. CP003699), suggesting these 2 clinical isolates may represent a novel genotype of *M. abscessus*, and that CGE may be a potential approach for subtyping this species complex.

**Figure 2:**
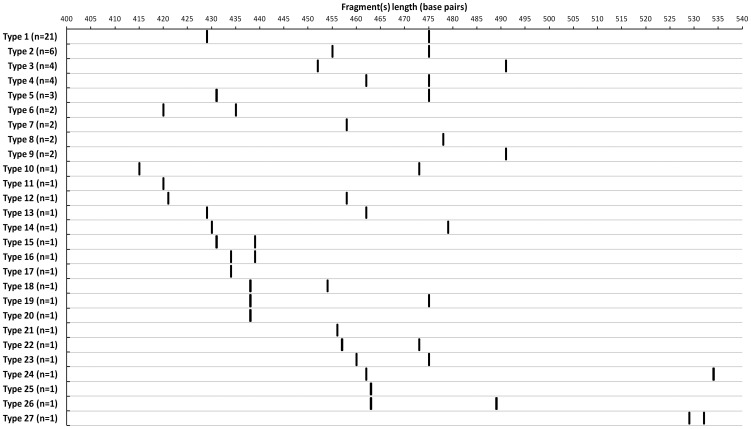
Fragment length heterogeneity within the *M. fortuitum* complex (n = 64) following CGE of the PCR product of the 16S–23S internal transcribed spacer. The number of isolates with each capillary gel electrophoresis type is indicated (*n*).

Amongst the 64 clinical *M. fortuitum* complex isolates, 27 different electropherograms were demonstrated. Five CGE types (CGE Type 1 to 5; Figure 2) accounted for 38 (59.4%) of the *M. fortuitum* complex isolates tested, with a high degree of heterogeneity seen with the remaining isolates (Figure 2). Clustering by geography or specimen type was not observed. Almost all (175/178; 98.3%) clinical isolates could be assigned to the correct species by the ITS-CGE typing technique based on analysis of the number and size of the amplified fragments. The single *M. moriokaense* isolate could not be speciated as it produced an electropherogram with a single peak at 478 bp which was indistinguishable from those produced by 2 *M. fortuitum* isolates (CGE Type 8). Unique peaks were identified for the remaining 11/12 (92%) RGM species studied (summarized in Table 2).

**Table 2:** Internal transcribed spacer fragment sizes determined by capillary gel electrophoresis for clinical isolates, including common fragments and unique lengths that characterise a species.

Species	No. of isolates	No. of CGE patterns	Single peak (%)	Common fragment sizes in bp (frequency)	Signature (unique) fragment lengths in bp
*M. chelonae*	40	1	100	384(14)/385(26)	384/385
*M. abscessus*	31	2	100	388.0(29)^a^	388.0/393
*M. massiliense*	17	1	100	388.5(11)^a^/389.0(6)	388.5/389
*M. fortuitum*	64	27	17	429(22)/462(7)/475(36)	415/420/421/430/431/434/435/438/439/455/456/457/458/460/462/463/473/475/479/489/491/529/532/534
*M. mucogenicum*	13	6	92	522(8)	332/372/516/518/521/522
*M. flavescens*	5	2	80	468(4)	468/477/506
*M. elephantis*	3	3	33	429(2)	463/480/498
*M. immunogenum*	1	1	100	400	400
*M. moriokaense*	1	1	100	478^b^	
*M. neoaurum*	1	1	0	490/505	505
*M. phlei*	1	1	100	461	461
*M. septicum*	1	1	0	467/493	467/493

a Rounding to 0.5 base pairs allowed for reliable separation of *M. abscessus* and *M. massiliense*.

b *M. moriokaense* could not be separated from two isolates of *M. fortuitum* with indistinguishable electropherograms displaying single peaks at 478 bp.

### Reproducibility

Reproducibility of the ITS-CGE technique was assessed by repeating the method on 19 (10.7%) isolates (with a total of 28 peaks) on different occasions with fresh PCR reagents. The average measured difference between the repeat fragment sizes was 0.12 bp (range of 0.0 to 0.39 bp). In addition, 3 patients from whom multiple clinical isolates were obtained during the study period, underwent comparison of CGE electropherograms derived from independent specimens. The average measured difference between the fragment lengths for the related isolates was 0.05 bp (range 0.01 to 0.08 bp).

### Antibiotic susceptibility

Antimicrobial susceptibility data was available for 19/29 (65.5%) *M. abscessus*, and 10/17 (58.8%) *M. massiliense* isolates. The only significant MIC difference was seen with clarithromycin, with *M. abscessus* strains having a median MIC of 1 mg/L (IQR 0.25–4) compared to *M. massiliense* median MIC 0.25 mg/L (IQR 0.25–0.25), p<0.01. Only a single isolate of *M. massiliense* had a clarithromycin MIC of >0.25 mg/L.

## Discussion

The reliable and accurate identification of RGM is challenging because of the lack of consensus in the current nomenclature as well as the absence of a robust and universal method for speciating all RGM. Traditional methods are labour intensive, time consuming and require the interpretation of subjective data [29]. Here we report a novel method of applying CGE to analyze the RGM 16S–23S ITS region, as a highly discriminatory and reproducible method for speciation of RGM. While other authors have utilized 16S–23S ITS restriction analysis [11], [30], [31] or probe hybrididization [26], this is the first study that has applied the high resolution CGE method to the intact ITS fragments and demonstrated it as a highly discriminating tool for the RGM group, including the delineation of the *M. abscessus* group. The method was initially validated using 19 ATCC strains, with 73.6% identified overall – 100% of RGM and 66.7% of slowly growing mycobacteria. When applied to clinical isolates of RGM the diversity of electropherograms generated allowed for the allocation to species of 98.3% of the clinical RGM strains studied.

A key finding of the present study was that the ITS-CGE method clearly separates organisms belonging to the *M. chelonae* and *M. abscessus* complex on the basis of a 3–4 bp difference of the rRNA 16S–23S ITS fragment length. Notably, the high resolution of the CGE method was able to characterize the *M. abscessus* group into *M. massiliense* and *M. abscessus* on the basis of three consistent ITS polymorphisms resulting in a 1 bp difference in fragment length. Previously it has been reported that single gene sequences were inaccurate in separating the *M. abscessus* group, although the 16S–23S ITS was not considered by these authors [32]. Our finding that the mycobacteria ITS is sufficient to separate these closely related subspecies is consistent with previous reports of the utility of the ITS sequence in separating both the slowly and RGM groups [10], [11].

The requests for clinical microbiology laboratories to delineate the *M. abscessus* group is likely to increase in the future with emerging evidence that sub-species within the group are associated with differing clinical outcomes [16]–[18], transmission within cystic fibrosis populations [33], and therapeutic implications, including the propensity for *M. abscessus* to develop macrolide resistance via activation of an intact *erm* gene [13]. This propensity to macrolide resistance is supported by the MIC data in our study population which demonstrated *M. massiliense* isolates have significantly lower MICs for clarithromycin (p<0.01). The single isolate of *M. massiliense* identified with an MIC of 16 may have harbored an alternate mechanism of resistance, such as point mutations of the 23S rRNA macrolide binding site [13], [34], or may have expressed a functioning *erm* gene as has been recently described in a subset of *M. massiliense* isolates [35]. Nevertheless, the susceptibility data presented here supports the emerging view that laboratories need to delineate the *M. abscessus* group given the predictable clarithromycin susceptibility data along subspecies lines.

Electropherograms with distinct double peaks were identified for some RGM complex and species groups (Figure 1, Panel D; Table 2), presumably correlating with multiple genomic copies of the ITS. These double peaks increase the discriminatory power of the ITS-CGE method. For example, a fragment length of 429 bp was seen for a proportion of the *M. fortuitum* complex isolates as well as for one of the *M. elephantis* isolates. However, in each of these cases, the electropherogram revealed a distinct and unique second peak allowing for differentiation of the species. This study has highlighted the 16S–23S ITS sequence diversity of the RGM group, particularly within the *M. fortuitum* complex. The degree of resolution naturally lends this method to subtyping within this species complex where a high degree of hereogeneity was demonstrated (Figure 2); however, the clinical utility of this approach in the tracking of patient isolates and in the investigation of nosocomial outbreaks, requires further evaluation.

Despite the excellent reproducibility of the ITS-GGE technique, electropherogram features need to be interpreted with clear criteria to allow consistent reporting [24]. Double peaks which were less than one bp apart were encountered in a small proportion of isolates which possibly arise due to variable secondary structure of the amplicons (see examples; Figure 1, Panel A and D). Sequencing of these fragments confirmed that the calculated ‘true peak’ consistently correlated with the larger peak. The accurate interpretation of these double peaks is especially important if using the method to resolve *M. abscessus* from *M. massiliense* as the distance between the ‘true’ unique peaks for these species (measured at 0.72 bp) may be less than the difference observed between the spurious double peaks. Variation was also seen in the relative height of fragments in electropherogram peaks, including some small peaks not analysed that were <10% of the highest peak. The relative heights remained consistent on duplication of isolates and may be attributed to the variation in genomic repeats of 16S–23S ITS region. The relative variation in peak heights (when 2 peaks are present) may provide additional power to discriminate between closely related species.

There are several important benefits of ITS-CGE method when compared with tradition identification procedures [22]–. The method can be completed within 1 to 2 working days and does not rely on time and labour-intensive agar gel techniques. Although complete cost analysis is yet to be performed, the ITS-CGE based method is less expensive (approximately $3 per isolate) compared to 16S rRNA sequence base method commonly employed for speciating the less frequently encountered species. The method has the capacity to distinguish novel species or subtypes which is in contrast to phenotypic and proteomic methods. The results generated by ITS-CGE are amenable to inter-laboratory comparison and allow portability across jurisdictions. Importantly, the method appears to be able to identify mixed cultures. For example, when we experimentally mixed *M. abscessus* and *M. fortuitum* colonies prior to extraction and then applied the ITS-CGE technique, the electropherogram contained the unique or ‘signature’ peaks for both species (Figure 1, Panel F).

Limitations of this study include the relatively low number of the less common RGM species analyzed. In addition, it is possible that intra-species genetic variation in isolates circulating in disparate geographical settings may be associated with variation in electropherograms. This was demonstrated by the unique electropherogram generated by the *M. fortuitum* ATCC 6841 strain when compared to the clinical *M. fortuitum* study isolates. As a result of this variation, laboratories which choose to implement this method will need to validate the findings in their own geographic settings. This limitation could be overcome if laboratories were to generate a collaborative CGE database, incorporating well characterized clinical strains from disparate geographical locations. Various commercial sequencing companies provide CGE which may be useful for laboratories that do not have access to the required equipment and expertise.

In conclusion, the amplification of the *Mycobacteria* 16S to 23S rRNA ITS region(s) followed by fragment analysis by CGE enables the identification of clinically relevant RGM. This study has demonstrated that the ITS-CGE method can distinguish the taxonomically related but clinically important *M. abscessus* and *M. massiliense* species, as well as discriminate the heterogenous *M. fortuitum* complex. With the development of a comprehensive CGE-ITS database this method may complement existing approaches in the identification and characterization of RGM.

## Supporting Information

Figure S1 Mycolic acid analysis by High-Performance Liquid Chromatography (HPLC) showing typical chromatograms for A) *M. abscessus*/*M. chelonae*; B) *M. fortuitum*; C) *M. flavescens*; and D) *M. mucogenicum*. * = low standard; + = high standard.
(TIF)

Table S1 Outlines identification method employed for the rapid growing mycobacteria clinical isolates included in the study cohort.
(DOCX)
