# Supplementary figures and images for: Improved Identification of Rapidly Growing Mycobacteria by a 16S–23S Internal Transcribed Spacer Region PCR and Capillary Gel Electrophoresis
Source: PLoS One. 2014 Jul 11;9(7):e102290. doi: 10.1371/journal.pone.0102290 (PMC4094492; doi:10.1371/journal.pone.0102290)

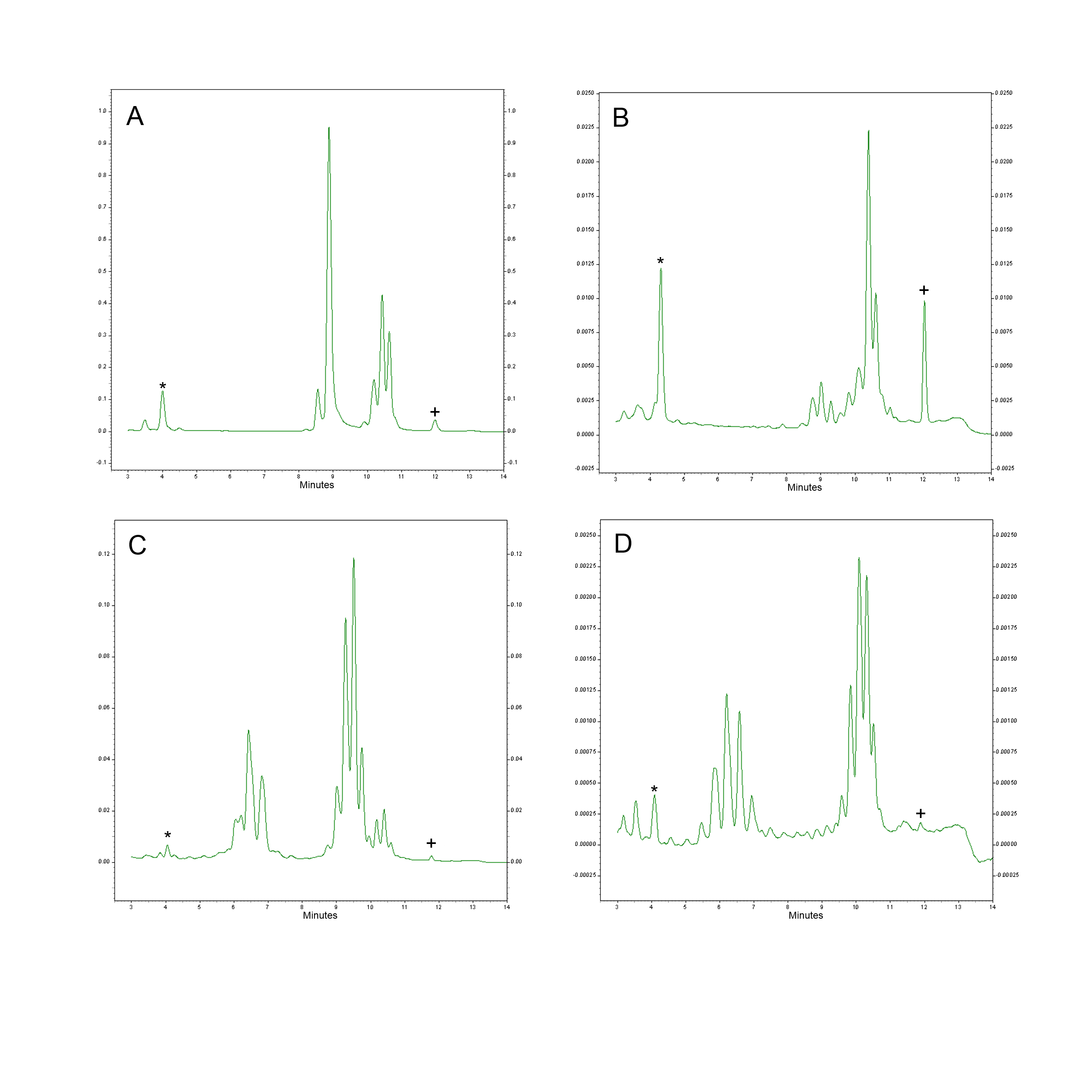

Supplement: Figure S1 — Mycolic acid analysis by High-Performance Liquid Chromatography (HPLC) showing typical chromatograms for A) M. abscessus/M. chelonae; B) M. fortuitum; C) M. flavescens; and D) M. mucogenicum. * = low standard; + = high standard. (TIF) [file pone.0102290.s001.tif]
