# Supplementary material for: Improved Identification of Rapidly Growing Mycobacteria by a 16S–23S Internal Transcribed Spacer Region PCR and Capillary Gel Electrophoresis
Source: PLoS One. 2014 Jul 11;9(7):e102290. doi: 10.1371/journal.pone.0102290 (PMC4094492; doi:10.1371/journal.pone.0102290)
Supplement: Table S1 — Outlines identification method employed for the rapid growing mycobacteria clinical isolates included in the study cohort. (DOCX) [file pone.0102290.s002.docx]

| **Species** | **No. of isolates** | **HPLC [25]** | **16s rRNA sequencing** | **16S-23S rRNA real time PCR [26] or sequencing** |
| --- | --- | --- | --- | --- |
| *M. chelonae* | 40 | - | - | 40 |
| *M. abscessus* | 31 | - | - | 31 |
| *M. massiliense* | 17 | - | - | 17 |
| *M. fortuitum* | 64 | 53 | 11 | - |
| *M. mucogenicum* | 13 | 6 | 7 | - |
| *M. flavescens* | 5 | 1 | 4 | - |
| *M. elephantis* | 3 | - | 3 | - |
| *M. immunogenum* | 1 | - | 1 | - |
| *M. moriokaense* | 1 | - | 1 | - |
| *M. neoaurum* | 1 | - | 1 | - |
| *M. phlei* | 1 | - | 1 | - |
| *M. septicum* | 1 | - | 1 | - |

HPLC – high-performance liquid chromatography
